# A typology of practice narratives during the implementation of a preventive, community intervention trial

**DOI:** 10.1186/1748-5908-4-80

**Published:** 2009-12-14

**Authors:** Therese Riley, Penelope Hawe

**Affiliations:** 1 Centre for Health and Society, Melbourne School of Population Health, The University of Melbourne, Level 4, 207 Bouverie St, Carlton, Victoria, 3010, Australia; 2 Population Health Intervention Research Centre, University of Calgary, 3330 Hospital Drive NW, Calgary, Alberta, T2N 4N1, Canada

## Abstract

**Background:**

Traditional methods of process evaluation encompass what components were delivered, but rarely uncover how practitioners position themselves and act relative to an intervention being tested. This could be crucial for expanding our understanding of implementation and its contribution to intervention effectiveness.

**Methods:**

We undertook a narrative analysis of in-depth, unstructured field diaries kept by nine community development practitioners for two years. The practitioners were responsible for implementing a multi-component, preventive, community-level intervention for mothers of new babies in eight communities, as part of a cluster randomised community intervention trial. We constructed a narrative typology of approaches to practice, drawing on the phenomenology of Alfred Schutz and Max Weber's Ideal Type theory.

**Results:**

Five types of practice emerged, from a highly 'technology-based' type that was faithful to intervention specifications, through to a 'romantic' type that held relationships to be central to daily operations, with intact relationships being the final arbiter of intervention success. The five types also differed in terms of how others involved in the intervention were characterized, the narrative form (*e.g*., tragedy, satire) and where and how transformative change in communities was best created. This meant that different types traded-off or managed the priorities of the intervention differently, according to the deeply held values of their type.

**Conclusions:**

The data set constructed for this analysis is unique. It revealed that practitioners not only exercise their agency within interventions, they do so systematically, that is, according to a pattern. The typology is the first of its kind and, if verified through replication, may have value for anticipating intervention dynamics and explaining implementation variation in community interventions.

## Introduction

Although there are established methods for tracking the delivery of health promotion and preventive interventions [1,2], the dynamic of what happens in practice still remains elusive [3]. Many large-scale community-level preventive interventions over the last 20 years have failed or have had very modest effects [4,5]. This has been attributed in part by many commentators to the fact that investigators rarely examine in detail what happens within the 'black box' of an intervention, complicated also by implementation reporting inadequacies [6-9]. Implementation science has thus emerged as a promising new field of investigation [10]. Understandings are needed that appreciate the complexity of the phenomenon, taking into account the sometimes vexed experiences of practitioners at the coal face of intervention implementation. In particular, the practitioner's viewpoint may be critical for illuminating theories of action that could strengthen intervention effectiveness [11].

This study set out to explore the experience of community development practitioners implementing a new community-based universal preventive intervention in maternal health. The paper begins by describing the research context and data collection methods. We then describe the construction of a narrative typology of community intervention practice. Such a typology could be used to anticipate implementation challenges (prior to or during) intervention implementation. It may also assist in the interpretation of intervention outcomes. We conclude by discussing the implications of the typology for practitioners and researchers interested in gaining greater insight into the dynamics of community health interventions.

## Methods

PRI SM (Program of Resources Information and Support for Mothers) was conducted as a large-scale community cluster randomized trial comprised of eight intervention communities and eight comparison communities (n = 18,555 women). The goal was to prevent post-natal depression and improve maternal health. The setting was Victoria, Australia. PRISM involved a range of primary care and community-based strategies [see [12]]. This included training for general practitioners and maternal and child health nurses, as well as information kits and initiatives facilitated by a local steering committee. Nine community development officers (CDOs) were employed for two years (one per intervention community, or in one case, two job-sharing in one community). The CDOs were the primary agents in PRISM's implementation in relation to the community-based strategies. The research presented here was undertaken as a part of an independent concurrent project called EcoPRISM. EcoPRISM was an economic and ecological evaluation of PRISM [13]. The EcoPRISM project obtained ethics approval from LaTrobe University, Melbourne, Australia (Reference number 00/100)

### Data collection

An agreement of the CDOs' employment was to document aspects of their practice for the EcoPRISM project. This included maintaining a field diary over the two years of the intervention's implementation. The use of diaries in research tends to fall into two broad categories. The first involves the analysis of existing diaries or journals written by individuals within a particular historical context, such as the diary of Anne Frank [14]. The second category is the use of 'solicited' diaries as a data collection method. A range of qualitative and quantitative analytic techniques are then applied to the analysis of such data (for a detailed description of the use of diaries in social research see [14]).The use of 'solicited' diaries have a strong tradition in disciplines investigating peoples behaviour over time. For example 'time use' diaries have been used in studies that investigate the way people allocate time to particular activities [15]. Patient diaries are commonly used in studies of adherence with medication regimes [16]. However, there is less of a tradition in the use of diaries or journals in studies of professional practice with the exception of education, where diaries or journals are used by students or teachers as tools for reflection [17]. The key features of a diary include: some 'regularity' to the entries 'over a period of time' [14]; diaries are personal, that is, written by one person [14]; they are written in a 'contemporaneous' manner [14], that is, written at about the time of an event; and diaries create a record [14]. This incorporates 'what an individual considers relevant and important. It may include events, activities, interactions, impressions, and feelings' [[14] p.2]. For the purposes of evaluating intervention implementation from the perspective of practitioners, a 'solicited' diary method was the most appropriate approach.

The diaries in our study aimed to capture the CDOs' reflections, feelings, and theories regarding intervention implementation. The diaries were either handwritten, electronic files, or emails. Some were a combination of these. The CDOs spent approximately 1.5 hours a week on documentation, including the field diaries [18], and the average diary consisted of approximately 40,000 words [18].The diaries were usually sent from the CDOs to the research team once a month. They ranged from a couple of entries a week, to once a month when the intervention work demanded all of their time [18]. There is conflicting evidence (from diary studies) as to the nature of 'respondent fatigue' [19] and whether diary entries and response rates decline over time. A study of the effects of communal gardening on the health of older people applied a diary method over approximately 23 weeks and found that through 'continued researcher support' respondent fatigue could be prevented [19]. This is consistent with our experience. The CDOs also maintained more traditional forms of intervention documentation that are described elsewhere [12,13]. Ongoing contact and support from the EcoPRISM research team over the two years of intervention implementation resulted in a data set that was rich with detail.

The field diaries remained confidential between the CDO and the EcoPRISM Research Fellow (TR). This agreement was extended to include an EcoPRISM co-investigator (PH) at the end of the two-year implementation period, with the consent of the CDOs. The restricted access to the data was designed to provide a safe environment in which CDOs could talk about the difficulties of implementation as well as the success stories. Periodic interviews with CDOs (34 in total) were also undertaken. These interviews provided an opportunity to capture the reflections of CDOs who were less comfortable with writing [19]. These were transcribed and included in the narrative data set to supplement what the diaries recorded. Primary analytic attention was paid to the field diaries as the prospective narrative data.

The result was a large data set (approximately 1,500 pages) outlining the challenges, triumphs, and personal pitfalls of implementing an intervention. Practitioners wrote descriptions of meetings and events; confessions of things going wrong; personal challenges of living and working in a community; explanations of success and fear of failure. They also provided detailed assessments of community infrastructure, identity, and culture.

After the first three months of data collection and analysis, an 80-page report was constructed that raised general issues for the PRISM research team to use in formative evaluation [20]. This also allowed the CDOs opportunity to see how their data and insights were being used and to comment on issues raised. After this, no formal progress reports were constructed. But, as diaries were read and interviews undertaken, any key points that might be of value for adjusting the intervention were raised within memos and in meetings between the EcoPRISM research team and the PRISM research team. The PRISM research team also monitored PRISM process and adjusted the intervention in response.

Our purpose was to not simply reveal details of what CDOs were doing, but to see if we could construct models of practice from the unique insight the data set provided. We collected and analysed this data abductively (rather than inductively) and, as such, our approach is based on the following epistemological assumptions [21]: our (social scientific) knowledge will be gained from the subjective meanings of the CDOs (gathered via field diary and interviews) and the concepts they use to understand their practice [21]; much of the CDOs' practice (and program implementation more generally) occurs in a 'taken for granted' or routine way [21]; and in order to gain access to the language or meaning given to their activities, we may have to use methods (such as diaries) to create the conditions for reflection [21]. Through the application of abductive reasoning, pre existing theory may be applied to provide added insights into the analysis of the meaning provided by the CDOs themselves [21]. We have drawn on a number of theoretical and conceptual frameworks to ensure our analysis is relevant to the research task at hand. This is consistent with an abductive strategy [21]. The analytic steps in this analysis are described below.

### Narrative analysis and individual narratives

Narrative analysis aims to uncover the underlying subjective meaning structures that form the basis of how people come to understand and evaluate the world over time [22]. The focus is not just on what happened but what is revealed by the way a 'story is told' [22]: the plot, the position of the characters [23], the enablers, and constraints. Narrative is an interdisciplinary research approach that has been used in illuminating experience of illness [24,25] and within a range of disciplines, such as education [26,27] and sociology [28,29]. Narrative analysis was therefore an ideal choice to get deep inside the private contexts of practice [30]. Alternatively we could have undertaken a thematic or content analysis of the diary data. However, this would have 'decontextualised' the data, as themes are carved out and analysed independently of the context of their creation [31] Thematic analysis also removes or downplays the significance of time [18]. Time is a critical component of a narrative analysis in two ways. First, it assumes that the past and the future come to bear on present experiences [32] and the decision-making of the CDOs. Second, by maintaining the vantage point of the one practitioner, we learn about their interpretations of events over time [22]

While many studies that undertake a narrative analysis draw on interview data, there is no epistemological reason as to why diary data are not conducive to narrative analysis [14]. As a method of data collection, the diaries allowed the CDOs to reflect on their own practice in their own words. They had greater control over what was written about or excluded [19]. This degree of control over the data could be viewed as a limitation of the method if undertaking a content analysis. That is, where the factual accuracy of the events recorded is paramount (for an example of the content analysis of diaries see [33]). However, a narrative analysis is less concerned with whether the events happened exactly as described and more concerned with 'identifying the structure which underpins specific narratives and the ways in which these structures enables the narrator to make sense of and present their lives' [[14] p.89]. It focuses attention on the interpretation of events rather than the events themselves [34].

**Figure 1:**
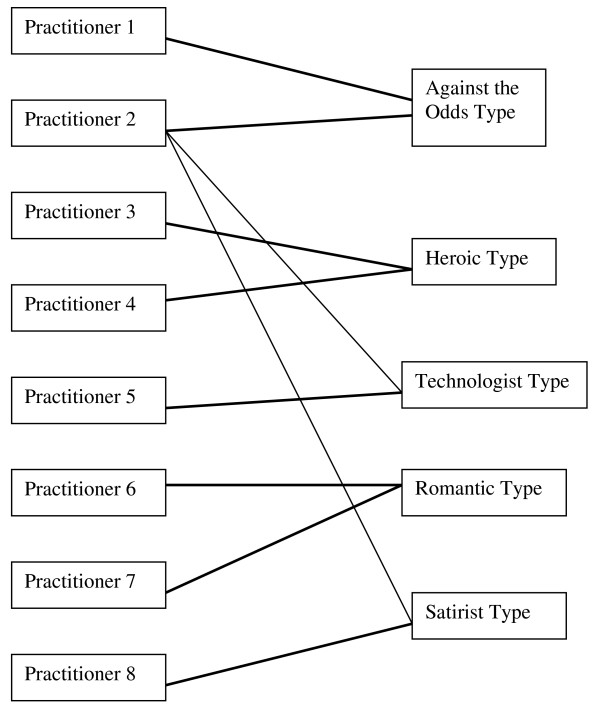
**The collapsing of individual narratives into a typology**.

Analysing plot structure is a common form of narrative analysis [31]. However, codifying narrative data is difficult [31]. The analyst cannot afford to decontextualise the data (as in thematic analysis) while looking for larger meaning structures that make up a narrative. The context in which a narrative is constructed becomes a part of the narrative itself [22]. So, coding was undertaken in two ways. Firstly, sensitizing narrative concepts such as whether the text was descriptive or evaluative in nature [35] were used to assist in applying a narrative lens to the data. Primary coding consisted of analytic notes attached to sections of text. These analytic notes comprised statements or evaluations of narrative structure and subsequent themes. A set of narrative questions/themes guided this analysis [18] such as how the practitioner position themselves in the telling of the story and the context of the story telling occasion [22] This approach to handling the data is consistent with the phenomenological aim of understanding the meaning people give to their lives in context and over time [32,36]. For a detailed description of methods see [18]. The two-stage analysis culminated first in the construction of eight individual narratives. Each practitioner narrative was then abstracted further into one of five types that make up the typology, as shown in Figure 1. Practitioner narratives were connected to a type if they encompassed many (but not necessarily all) of the characteristics of the type. Practitioner two was the only exception, encompassing key characteristics of three types.

### The rationale and development of a typology

Our interest is in abstracting meanings relevant to understanding 'course-of-action' [37] within a confined context. The result is the construction of a model or abstract description [21] of community-level intervention practice. For the model constructed in this study, we use the label of typology because it is comprised of a taxonomy of types of practice. In our case, the typology consists of a series of 'ideal types'. An ideal type is a technical term developed by Max Weber that refers to the creation of an analytic construct built from various aspects or characteristics of a given phenomenon [38]. Alfred Schutz who built on Max Weber's theory contends that ideal types can be thought about in two distinct ways. The 'personal ideal type' refers to a person who communicates [36]. The 'course of action' ideal type refers to the 'expressive process itself' or the product of that process [[36] p.187]. In other words, it is the practice, rather than the practitioner, that is the focus of our study. The CDOs' practice is a combination of action and events and the narration of such action and events

While we draw on Weber's 'ideal type' theory, for the sake of simplicity we will hereafter use the term 'Type' (with a capital T) when referring to an ideal type. Max Weber never intended the term 'ideal' to refer to a 'morally superior' way of acting [39], and we want to avoid any confusion for the reader. This is not a study of best practice. The construction of explanatory models or typologies from qualitative data is evident in a range of studies by scholars in other fields. For an example of the construction of ideal type narratives from interview data see [29]. For an example of the construction of a typology from interview data see [40].

According to Weber's, theory, a constructed type, while containing no falsehoods, 'contains no *particular *statements of fact' (emphasis ours) [[38], p. 90] because it is not constructed to represent customary expectations or to test micro-predictions in the immediate sense. Rather, its logic involves the surfacing of fixed relationships among phenomena of interest that allow the analyst to see a meta-theme that might not otherwise be observed [38].

Linguistic typologies, for example, study and classify languages according to their structural features. The typology allows the linguist to trace higher order and historic patterns that inform, for example, our understanding of the spread of human populations. But to know the meaning of any particular word, a linguist still uses a dictionary. In the same sense, our intention was to construct a typology that would alert researchers and practitioners to dynamics and dimensions that might be missing in the methods for implementation evaluation used traditionally. But there are numerous methods to know precisely what people do and when in a community intervention, such as event logs kept by practitioners [1] and observational monitoring by third parties [41]. These were also methods used within EcoPRISM [13].

### Determining the attributes of the typology

We have drawn on narrative literature to identify critical characteristics of a narrative, such as the plot (organising theme) [34] and characterisation of the 'supporting cast' [23]. Attributes one to six are drawn from this literature [22,23,32,34,42,43]. The seventh attribute comes directly from our research objective in understanding community-level intervention practice. We have deliberately focused analytic attention on the social contexts of practice and how practitioner agency is defined. By 'agency' we mean the nature of practitioner 'action' [44].

#### 1. Organising theme

The 'organising theme' creates a consistency in how the practitioners evaluate situations or events [34]. The 'organising theme' (or plot) is at the heart of a narrative [34]. This may be a metaphor or a strategy of some sort. For example, restitution is the 'organising theme' [34] for a narrative developed by Frank [24] to describe the way in which some people come to understand their illness as a journey back to the health they experienced prior to illness. The 'organising theme' [34] transcends the actualities of daily life. By looking beyond these activities or events we learn something of what motivates the protagonist.

#### 2. Narrative form

By narrative form we mean the temporal flow of a practitioner's evaluations of the interventions implementation over time [23]. We have drawn on literary theory to add depth to our understanding of plots, such as comedy or tragedy [42,43]. Other qualitative studies have also drawn on these types of narrative form in their analysis of narrative data [28].

#### 3. Protagonists position

The protagonist in this analysis refers to the practitioner. The protagonist's position is where the practitioner positions themselves in their narration. For instance, a practitioner may be the chief protagonist of their narrative [23]. Or, they might cast themselves more as an observer than the chief actor.

#### 4. Characterization of the supporting cast

This refers to how a practitioner may characterize the people they incorporate into their narrative. Gergen and Gergen [23] refer to these people as the 'supporting cast'. While there are likely to be similar people or organizations within the practitioner's narration, the constructed types differ on how these people or organizations are interpreted. These interpretations of others feed into the practitioners views on what to do and how to act [23]. The supporting cast [23] might be described only when they cause conflict and trouble, for example. Or they may be reported on throughout as supporters of the protagonist. They might be given roles as interpreters of events.

#### 5. Position or role of the audience

Within a narrative the audience (or listener) is an important part of the process of story telling [22]. The position or role that the audience is ascribed reveals something of the motivation of the practitioner. The audience may serve a legitimizing function as the practitioner seeks understanding from the audience. The audience may also be asked to challenge convention as they are made aware of alternative interpretations of events. The CDOs were aware that they were writing a diary that would be read and analysed by the EcoPRISM researchers, but did they take the trouble to explain or pardon their actions to the audience? Did they appear to seek approval or sanction? Or was the audience largely ignored?

#### 6. Resolution in context

This refers to a narrative's 'valued endpoint' [42]. The resolution of a narrative is value laden and interwoven with the other attributes of the typology. Resolution of the narrative is closely tied to the organising theme [34] and form of the narrative [23]. Did a story end on a high point or a low point? Did it appear to stop midstream and unresolved? The final diary entries reveal how a practitioner comes to understand or evaluate their time as an intervention practitioner. However, the resolution of a narrative does not equate to the ending of the intervention. This is an important point. The CDOs completed their final diary entries when their employment on the intervention ended. This was a time of uncertainty within the overall PRISM project.

#### 7. Orientation of their practice

This refers to the contexts of practice and the type of agency the practitioners embrace in order to effect change. In other words, this refers to where and how practitioners create transformative change in their community. For example, a practitioner may spend most of their time working within organizational settings influencing policy development. Alternatively they may spend their time raising awareness of an issue within a broader community setting in the hope that community members will lobby local authorities for change.

### Checking the plausibility of the typology

An abductive logic was applied to the construction of the typology and refers to the process of drawing on peoples descriptions of social life to create social scientific description or explanation [21,45]. This is like the chemical process of distillation to uncover the true essence of meaning. If the process has worked, the result should be a model of 'typical courses of action' in typical contexts by typical people [45]. In other words, similar practitioners placed in similar intervention contexts with similar characteristics should behave similarly. This is what Schutz refers to as the 'postulates of adequacy' and 'logical consistency' [37]. To test the plausibility of the model, we engaged in presentations and dialogues with research participants, other practitioners, and researchers.

The typology was constructed after the CDOs completed their employment. The narrative analysis of data was then undertaken over 12 months and from this the typology was constructed.

## Results

### Characteristics of the five types

Table 1 presents the characteristics of each of the five constructed types according to three of the seven attributes that make up the typology. The full typology showing all seven attributes can be accessed as an additional file to this manuscript (see Additional File 1; Table S1 - A typology of practice in community level interventions). Each of the types set out with the same goal of implementing the PRISM intervention as defined by the PRISM investigators. Yet, the narrative types differ markedly on how they characterize the key players [23], the kind of organising theme [34] that underlies their understanding, and the manner in which they orient their practice.

**Table 1:** Extracts from the typology of practice in community level interventions (illustrated with respect to three of the seven attributes*).

	ATTRIBUTE		
**Type**	**Orientation of Practice**	Characterization of the supporting cast**	Resolution**

The Romantic Type	Practitioner expressed agency in nurturing and maintaining relationships. It is in the context of personal relationships that change takes place.	Understood according to personal qualities. They are positioned in the narrative according to the role or function they serve within the relationship.	Happy ending if relationships are intact.

The Heroic Type	Orientation to the future. Work inside and outside conventional settings. Values the agency of individuals to create change.	A moral positioning according to roles, with a particular focus on 'blocking characters'. Utilitarian approach to relationships.	The re-distribution of power.

The Satirist Type	Orientation to the future. Work within conventional institutions. Agency is expressed through the analysis of situations.	The supporting cast is rarely taken on face value. Their character is assessed according to careful observation.	No satisfactory resolution. We don't know if the future predicted by the practitioner is realized.

The Technologist Type	Practitioner defers power to the intervention technology and works within institutional and managerial contexts.	Characterized according to role or function in the delivery of the intervention technologies.	Resolution suspended until evaluation results are known.

The Against the Odds Type	Practice focus is process. This process is applied through relationships. The practitioner is a facilitator of change.	Characterized according to community development logic., i.e., as people to be facilitated	The 'invisible fate' *i.e*., possible intervention failure, becomes visible to the practitioner.

*the full typology showing all seven attributes can be accessed as an Additional file (Table S1. A typology of practice in community level interventions)

** based on Gergen and Gergen 1984, 1988, Frye 1957, Ezzy 1998, Frank 2000

### Illustrations

We illustrate some characteristics of each of the types according to the key attributes of the typology, and in doing so highlight the typology's overall heuristic value. Greater detail about each type will be presented in later papers. The quotations presented here have been derived from the analytic notes drawn on to develop the individual narratives that were then abstracted into each of the types. All identifying information has been removed from the quotations.

### Contrasting roles of agencies and people

The contrasting roles played by agencies and people in the intervention provide our first illustration of the types. The Heroic Type, takes the narrative form of a heroic comedy [43] and the hero is the practitioner, the hero alone is responsible for the intervention outcomes. Other agencies and people are usually characterized as blockers [43] The 'blocking characters' create obstacles for the hero [43]. This forms 'the action of the comedy, and the overcoming of them, the comic resolution' [[43], p.164].

Blockers are framed as such because they have power [43]. This may be expressed in control of resources, decisions, or discourses. Senior managers are examples of people in control of resources. Researchers are examples of people in control of discourses. The CDOs who illustrate characteristics of this type spend considerable narrative space within their diaries discussing blocking characters [43], their impact on the CDOs practice, and ways of working with or around them. Blocking characters [43] occupy a moral space in the narrative type, as the following quotation illustrates. The hero's feelings are central to the story:

'Hi [EcoPRISM name], I haven't written anything for ages towards the journal -- have felt pretty snowed under. I have just had a meeting with [manager] this morning -- need to debrief with someone -- [the manager] has this way of making me feel as if I have totally lost my ground -- she turns arguments around and then has a way of putting the other person down -- all I seem to be able to do is defend my position. It's a mind [expletive]! A few months ago she was giving me messages about demanding too much from her and now I get messages about not giving her enough information.' (The Heroic Type: seven months into the intervention)

In this example, we hear about both the blocker and the blocking tactics of a manager delivering mixed messages about their relationship with the CDO. This is a field diary extract from the first seven months of the intervention. Over the next six months, this practitioner develops new strategies to manage the blocking tactics of the manager as the following quotation highlights.

'We don't tell her things yet, you know, because you're just never exactly sure what the political flavour of the thing is and she can just kill things so easily. So it's come up time and time again you know, 'Don't say it like this, say it like that...' It's just fascinating.' (The Heroic Type: eleven months into the intervention)

In contrast, with the Satirist Type, other people feature in the story centrally, but in ways less directly connected with the protagonist (the practitioner). The story is played out, not with the practitioner's actions, but in his/her appraisal of the roles of others in bringing about the intervention success or otherwise. This does not mean that the practitioner obviates all agency in the action. Rather, there is social distance in how the action is described -- less engagement, more observation, and heavy use of wit and irony to demonstrate that the 'truth,' or how things really are, is known only to the practitioner [43]. Rather than active blockers (the hero's narrative), what confronts the satirist is the apathy of people who stifle action. These people embody apathy and institutions allow it to exist via their conventions. These are key characteristics of a satire [43]. The status quo thwarts the implementation of the new program or policy as the following quotation highlights.

'... and there's always some you know, kind of unnamed bureaucratic reason why you can't do something new because the natural position of the bureaucracy [is] to say um, 'better not do something new really', and that's really what I was getting and he was a perfectly nice man.' (The Satirist Type: ten months into the intervention)

Character assessments are the vehicle through which the audience is made aware of barriers to intervention implementation.

' [Manager] rings me back. Her enthusiasm is distinctly controlled. Arrange meeting time. Even if her agency doesn't want to be involved, it's important politically not to put their noses out of joint, so I'll go optimistic and positive, and hope she picks up some positivity of attitude if not of commitment. I'll let her tell me how much they're doing, and what I should be doing.' (The Satirist Type: two months into the intervention).

The Satirist Type ridicules people who claim to be supportive and empathic to the goals of the intervention, but act inconsistently with this. At the same time, the satirist plays the role of 'letting' those people maintain that façade. The audience/reader is briefed on the situation instead, a situation that enshrines the satirist in a 'wisdom' role. It is also a passive role. One gets the sense that the hero is 'out there' risking life and limb to make the intervention work, whereas the satirist is taking care of the interpretation of who will bear responsibility or be blamed. That does not mean at all that the satirist is a saboteur or a pessimist, only that the satirist sees the intervention as simply one event in the greater scheme of things and interpreting that scheme is the substance of the narrative.

### Contrasting orientations to practice

The Romantic Type believes that it is in the context of personal relationships that social- and community-level change takes place. As a result, they spend their time nurturing and maintaining these relationships. This is achieved in the following example through time spent meeting people and gaining local intelligence

'So, it was an exhausting day, but very useful. I wonder whether I need to meet in person with everyone that approaches re PRISM. It's the way I work I guess, but it does make it tiring, but I feel like I've had the chance to build a rapport with these women...' (The Romantic Type: two months into the intervention)

The Romantic Type measures the quality of their work by the quality of these relationships. The Romantic Type prioritizes relationships over and above other tasks of intervention implementation. In the following example, the practitioner sensed they were behind in tasks associated with the development of intervention components, due to the time spent talking with mothers groups and generally building relationships in the community. As the quotation highlights, the practitioner was pleased, once expectations for assembly of key resource materials for PRISM were clarified, that she had not prioritised those tasks and made promises to the community she couldn't keep.

'... I had been worried about being behind [the other CDOs] in terms of signing and sealing vouchers, and compiling the information directory. I knew I had spent more time on general community development, and less on finalising those specific tasks. After Friday [when the PRISM research team clarified what was required in compiling the vouchers and information directory] I was glad I'd gone about things that way. Because some of the clarifications helped define more precisely what we should concentrate on in the vouchers and info directory, I felt that had I moved quicker on these I might have barked up the wrong tree, made promises I couldn't fulfil, got myself into a few sticky corners. A good community development worker will always find a way around this of course [if] it happens but since timelines are tight, I'm glad not to have to adjust, trim and backtrack at this point.' (The Romantic Type five months into the intervention)

In this example, the CDO is relieved that they don't have to break promises made in the community in order to meet tight deadlines. Relationships can remain intact. The pre-eminent place of relationships within this narration continues even when it becomes apparent to the CDO that key local players in PRISM's implementation may not truly understand the intervention, as the following quotation highlights.

'Yesterday I had coffee with [two nurses] and while very cordial and great fun, I came away with the depressing feeling that neither of them really understands what they're doing with PRISM, that they see PRISM as just another demand on their time, as something they have to do. I sympathize...' (The Romantic Type: seventeen months into the intervention)

The Romantic Type's way of orientating their practice is in stark contrast to the Technologist Type who values the directives of management and their own capacity to comply to expectations such as deadlines. The Technologist Type understands the intervention to be a series of core elements to be implemented locally and integrated into local institutional settings. These form the settings for practice. Power is deferred to the technologies of the intervention. This deference of power results in an adherence to the technological components of the intervention. Compliance with instructions, deadlines, and requests from the PRISM research team take up considerable narrative space. This is a consistent evaluative position from start to finish, and is representative of a stable narrative form [42]. In the following quotation, we sense the frustration with other CDOs who don't seem to value meeting deadlines in the same way as the Technologist Type.

'I haven't received many (1) Service Directory drafts from the other [CDOs]. The first week of March is over (nearly). Maybe they are working on a different time line to me? Am I losing the plot?' (The Technologist Type: four months into the intervention)

Similarly, in the following example the practitioner is working out what role the local steering committee will play in intervention implementation. Her default position is to look to the original instructions and if that is not clear then to contact the PRISM research team for direction.

CDO:....' I'm still trying to work out what we'll do at our first meeting and then trying to, I guess, work out what their goals are. I know it's all documented about what their role is but I suppose having a clear idea of what sorts of things they'll do, and I need to know that for them to know what to do of it, yeah. That's still a bit like mud at the moment, yeah.'

Interviewer: 'And how are you going to clear that up?'

CDO: 'Oh, I don't know, ring up the PRISM team and get them to help me. [laughter] I'll ring them up and ask them, yep.' [laughter] (The Technologist Type: two months into the intervention)

In deferring power to the intervention technologies, the Technologist Type acts as a conduit for the values and principles that are embedded in the design of such technologies [46]. The technological voice of program rhetoric permeates the following example as the practitioner identifies a possible source of tension between the local community views and those of the PRISM intervention.

'Conversation with [a PRISM coordinator] re [support] group illustrated some tension between local expectations/views and PRISM aims re what PRISM is about, [it] was useful to have discussion with [a PRISM coordinator] re PRISM perspective!' (The Technologist Type: seventeen months into the intervention)

Whereas for the Romantic Type power is exercised and gained in relational settings, for the Technologist Type change is dependent on the quality of the intervention technologies.

### Contrasting narrative resolution

An important feature of the typology is that regardless of the clearly specified PRISM elements and the common pre-trial training, guidance, and coaching by the PRISM research team, the five types differ as to how their narrative is resolved. Implementation of the intervention is not an endpoint in itself.

One of the most striking contrasts is between the Against the Odds Type and the Technologist Type. The Against the Odds Type takes the narrative form of a tragedy [23]. It is initially progressive. The practitioner is positive and hopeful in what can be achieved during implementation. This is followed by a regressive slide as the desired ending slips away from the practitioner's grasp [23]. The practitioner in the Against the Odds Type is a 'tragic hero' whom we understand to be great in their knowledge of the principles and logic of community development, someone who mediates between us (the audience) and what could be described as an 'invisible fate' [43]. In other words, as the Against the Odds Type describes the obstacles and barriers to intervention implementation, we are made aware of possible intervention failure. In the following example, a practitioner explains that difficulties with local management shouldn't necessarily be viewed as a lack of success. However, for the audience/reader one is left wondering how a short (two-year) intervention can succeed amidst such strong opposition.

'Actually I'd just like to say that I don't necessarily judge what's happening with [manager] as .... unsuccessful .... from a CD [community development] point of view, that can be a symptom that you're actually being quite effective and that you're pushing people's buttons because when you create change, you do.' (The Against the Odds Type: ten months into the intervention)

The Against the Odds Type comes to realize that their goal to instil in others the knowledge of community development was doomed from the beginning. The barriers were too great, and this is how the narrative is resolved.

' [It] [h]as been a long haul and it seems to me that it's really important from a CD perspective that when change is being implemented that the people who want the change (*i.e*., in this case [municipal] council) must be integrally involved in the process, at the coal face .... it was quite obvious now, looking back, that the changes PRISM wanted to focus on were so much of a paradigm leap for the person allocated to support the changes (*i.e*., the provider [the Maternal and Child Health Service]) that it would have made much more sense strategically to have moved this role to someone at [municipal] council .... I did suggest [this idea to the PRISM research team] but [I] was supported to keep trying with the [the prescribed PRISM approach].' (The Against the Odds Type: twenty three months into the intervention)

The Against the Odds Type resolves its narrative, without reference to the objective evidence to be presented by the trial team at a future date. This is in contrast to the Technologist Type for whom resolution is suspended until the health outcomes are known. This is because the Technologist Type defers power to the technologies of the intervention, including its outcome evaluation. For the Technologist Type, personal goals or opinion are secondary. The following quotation from the final interview with a practitioner is a good illustration of the separation of practitioner with personal opinions, goals, and expectations and the intervention technologies.

Interviewer: 'And what about your expectations, as the project worker?'

CDO: 'Expectations?'

Interviewer: 'Your expectations in the beginning about what you were going to get out of it, and whether or not they were met?'

CDO: [long pause] 'I don't know what I think I'd get out of it personally.' (The Technologist Type: final month of the intervention)

## Discussion

The CDOs were employed for their community development expertise, but they were placed within a project with fixed goals and fixed intervention components. The randomised trial design created a high stakes, highly scrutinized environment. So this is not a typology of unfettered community development practice. But it is a common scenario. Community development practitioners are often employed on projects not where communities lead, but where communities are invited to participate, collaborate, and strengthen an approach conceived originally by external researchers and/or funding agencies [47].

We believe CDOs gave us rich, authentic accounts of their work, because the diaries were confidential and not read by their managers (PRISM research team), and because results were confirmed by CDOs and a range of community practitioners in subsequent presentations and dialogues. We appreciate that in the early stages CDOs might have been tempted to write what they supposed we might like to hear. But to keep this going for two years would have been difficult. Plus, if CDOs were writing what they thought we wanted to hear, we would have expected more consistency across the writers in keeping with the traditional view of what the intervention was supposed to be. As it was, the practices described were diverse, in spite of the intervention having a standard form.

It is not unusual to use narratives to understand and represent community development processes [48,49]. But the extensiveness and intensiveness of this data set are unique. Plus, the phenomenological approach to analysis permitted a meta-synthesis with unique insights. So what have we found, and what do we make of our findings? Research in professional practice in medicine, nursing, social work, education, and the arts has studied decision-making [50], skill development [51], competence [52], sense-making [53], and management of uncertainty [54]. Sheppard and Ryan [55], for example, describe how social workers act as 'rule-using analysts' in everyday practice to analyse patterns, form hypotheses, and revise actions. Others have drawn attention to how both health professionals and practitioners in the creative arts use improvisation as an essential form of action. Farmer [[56], p.1] goes further to suggest that improvisation and theatre form the central metaphor of community development itself, arguing that community building is essentially about 'putting people together to create new conversations, new alliances and new possibilities.'

This paper represents, to our knowledge, the first intensive investigation of the role that community practitioners themselves play. It is all the more important because community development practitioners typically eschew the limelight. So anxious are they to see others take credit for project success, they rarely describe or own their particular contribution. In tracking and analyzing their narratives over time, what emerged was a typology not of best practice necessarily, but of real practice, the positioning of people and 'the stakes' among them. Later papers will elucidate the types and their distinctions in more detail. But it is important in the first instance to consider the overall structure of the typology, its parameters, and what we feel it offers.

Five criteria have been suggested to justify whether a typology is adequate [57]. These five criteria are: Is the phenomenon to be classified adequately specified? Is the classification characteristic adequately specified? Are the categories mutually exclusive? Is the typology collectively exhaustive? And finally, is the typology useful?

The phenomenon we describe is the unfolding of a community intervention through the chief facilitator's thoughts and actions. Our types are adequately specified if they are specific and coherent on replication. To verify this, we invite researchers in other contexts to embark on similar inquiry and see if the five types emerge. In the meantime, is it encouraging that the types encompass and echo the power struggles and cultural complexities that have been noted by others who have researched implementation processes [58] and those who have studied change agents [59].

Adequate specification of the classification characteristic refers to the columns in Table 1 and Table S1 (see Additional File 1; Table S1 - A typology of practice in community level interventions). Do the types truly pivot around these points? We acknowledge that researchers using other theories, such as activity settings analysis [60], would articulate practice in terms of roles, symbols, and relationships interacting over time, for example. We have been informed by this thinking and have already used the same data set to identify episodes of practice that conform with Kelly and Trickett's ecological theory [61]. But what our typology uniquely adds, that replication could potentially verify, is the overarching moral position and subjective meanings that provide the interpretative framework for all action. The organising theme [34], the orientation to practice, the narrative form [23], and the resolution [42] especially show this. Practitioner values are rarely incorporated into the frameworks used to interpret people's work, let alone the dynamics of how interventions unfold in communities.

Our categories do not set out to be mutually exclusive in the sense that they are not people types, but practice types. To test if the typology is collectively exhaustive, a replication study could test if other types, beyond our five, emerge. However it is quite possible that different contexts, cultures, and times might nuance these practice types differently. The value is therefore not simply in the replication of the types *per se*, but in the cross-validation by the practitioners themselves in the execution of the final most important criterion [57] - the assessment of the typology's usefulness.

The construction of archetypes has been used in other fields, like management science, to examine simultaneous associations among a large numbers of variables in order to yield higher order patterns that can be used in planning [62]. In the first instance, we suggest that the usefulness of our typology lies in the invitation to reflect on aspects of practice that might hitherto have been mostly unseen. Of all, the Technologist Type narrative is possibly the most familiar. This is because it is the one that many community intervention researchers have constructed previously, not with open qualitative methods as we have here, but with alternative 'identity-kit' means such as process monitoring tools and tracking devices closely tied with the delivery of intervention components or other prescribed specifications [41]. The Technologist Type is the one most associated with intervention fidelity as defined in traditional ways (*i.e*., conforming to a particular standardised technology). Indeed, the remaining four types are perhaps the worse nightmare of intervention researchers who ascribe to a conventional view of intervention integrity.

The insights from the narrative analysis go beyond the roles practitioners play as change agents, such as catalyst, solution giver, process helper, or resource linker [63], to reveal the deeper values guiding the way they make decisions. The narrative types invite new considerations of intervention dynamics, in particular the strengths and vulnerabilities of different ways of working. For example, the Romantic Type invites designers of community interventions to value and to harness the rich pre-existing relationships in communities. The potential down side is that preservation of relationships and upholding their development will cause the Romantic Type to stall on project deadlines and trade off on other priorities. The strength of the Heroic Type is commitment, dedication, and personal investment. The vulnerability is burnout and neglect to build sufficient significant responsibility taking in others. The strength of the Satirist Type is the acute insight and macro-level analysis that foresees how events will unfold before they happen. The vulnerability is disengagement and the sense of not feeling sufficiently valued. Getting the analysis correct may become more important than making the intervention successful. The strength of the Technologist Type is the adherence to the standard intervention protocol and the assurance of intervention fidelity traditionally associated with this. The vulnerability is the reluctance to improvise and adapt in ways that could strengthen intervention potency. The strength of the Against the Odds Type is the energy and commitment to 'textbook' style community development principles. The vulnerability is the possibility of scant allies on the ground with the same commitment and worldview. If it truly takes a village to raise a child, the task is very big indeed.

We see these types as products of the interaction of the context, the past experience and expertise brought by the CDOs, and the phases of action. So, we would be concerned if the construction of practice types from carefully observed experiences in community interventions was used to privilege something intrapsychic, such as personality theory, or worst, to suggest or assist those who might think that screening and selection of staff become formulaic (*i.e*., 'we'd like to recruit two Heroes Types, one Romantic Type, and as many Technology Types as we can get'). First, our types were rarely entirely embodied singly within one person. Second, we do not know from this analysis which type was associated with intervention success (and the trial sample size precluded this degree of fine discrimination being possible anyway).

To the practitioner, the value of the typology is the opportunity to own the personal in the professional, that is, to interrogate one's own position at any particular time point and either adjust it or embrace it consciously - to articulate why he/she believes certain actions have more benefits than others. Many of the types were susceptible to disillusionment in this community intervention, with its high expectations and uncertain, year-to-year funding. A tool for reflection and action could help. To researchers and theorists, the value of the typology is the acknowledgement it gives to the neglected concept of agency in community interventions, and to the diversity of agent roles and positions. Our research has illuminated that the agency exercised by practitioners forms five distinctive patterns. Within these patterns there are smaller stories about actions, strategies, causes, and purposes for doing one thing and not another [18,61].

Paul Valery, the French philosopher and poet, argued that 'there is no theory that is not a fragment, carefully prepared, of some autobiography' (Valery, cited in Olney, 1980)[64]. This statement was designed to draw attention to the theorist in every theory. Here our focus on the person is more flagrant. We have drawn on autobiographic methods from social science to create a picture of what happens in community intervention practice that is rarely seen in the process methods, impact logs, outcome surveys, and controlled designs that are usually constructed in community intervention research [5]. Having 'outed' this level of understanding and subjective meaning, the full implications are still to be confronted. To truly make complex interventions more successful, and to bridge the policy and program effectiveness gaps so often lamented [65], this new layer of meaning must be further explored.

### Epilogue

The typology of practice was constructed prior to the results of the PRISM intervention being made known to the authors, the CDOs, or the public. A paper published in 2006 [66] showed no effect of the PRISM intervention on maternal health at six months after birth. The results of the follow-up two years after birth have not yet been published and are unknown to the authors of this paper.

## Competing interests

The authors declare that they have no competing interests.

## Authors' contributions

TR conceived the idea, designed the field diary data collection protocols, undertook the interviews, analyzed the data, constructed the typology, and drafted the results. PH participated in the analysis of the data through critical appraisal of individual narratives and in the construction and interpretation of the typology. Both contributed to the drafting of the manuscript.

## Supplementary Material

Additional file 1 **Table S1. A typology of practice in community level interventions**. This table presents each of the five constructed types according to all seven attributes of the typology. Click here for file
