# Supplementary material for: A typology of practice narratives during the implementation of a preventive, community intervention trial
Source: Implement Sci. 2009 Dec 14;4:80. doi: 10.1186/1748-5908-4-80 (PMC2803442; doi:10.1186/1748-5908-4-80)
Supplement: Additional file 1 — Table S1. A typology of practice in community level interventions. This table presents each of the five constructed types according to all seven attributes of the typology. [file 1748-5908-4-80-S1.DOC]

**Additional file 1; Table S1.**

**A typology of practice in community level interventions**

| **Type** | **Organizing Theme*** | **Narrative Form*** | **Protagonists Position*** | **Orientation of Practice** | **Characterization of the supporting cast^*^** | **Position of the Audience^*^** | **Resolution^*^** |
| --- | --- | --- | --- | --- | --- | --- | --- |
| The Romantic Type | Relationships | Romance. This is characterized by a series of struggles that the practitioner overcomes that leads to success. | Relationships are the central character of which the practitioner is a part. | Practitioner expressed agency in nurturing and maintaining relationships. It is in the context of personal relationships that change takes place. | Understood according to personal qualities. They are positioned in the narrative according to the role or function they serve within the relationship. | The audience is directly involved in the personal drama, as they read about the relationships between people and events. | Happy ending if relationships are intact. |
| The Heroic Type | Strategic action | Heroic Comedy. This is characterized by the practitioner moving towards a happy ending by overcoming a significant barrier. | Practitioner is the central character. Practitioner is responsible for intervention outcomes. | Orientation to the future. Work inside and outside conventional settings. Values the agency of individuals to create change. | A moral positioning according to roles, with a particular focus on ‘blocking characters’.  Utilitarian approach to relationships. | Audience directly engaged in the drama. We are told of the strategies of ‘blocking characters’ and of the great personal risk to the practitioner. In doing so the audience does not know if the practitioner will succeed until the very end. | The re-distribution of power. |
| The Satirist Type | Others and their role in the intervention | Satire. This is characterized by observation and judgement about the role others play in the action. Wit and irony are used to point out the challenges of the present. Questions are posed about future success and who will take responsibility for it. | The practitioner is a commentator, a role that distances him/her from the action and consequences. | Orientation to the future. Work within conventional institutions. Agency is expressed through the analysis of situations. | The supporting cast is rarely taken on face value. Their character is assessed according to careful observation. | The audience is central to the narrative. They are made privy to the practitioner insights of what is really going on. | No satisfactory resolution. We don’t know if the future predicted by the practitioner is realized. |
| The Technologist Type | Compliance with the values and principles of the intervention technologies. | Stability. This is a narrative form in which a practitioner’s evaluation of situations and events remains the same over time. | Central character is the intervention technology. Practitioner is an agent of the technology. | Practitioner defers power to the intervention technology and works within institutional and managerial contexts. | Characterized according to role or function in the delivery of the intervention technologies. | Audience is a passive observer.  How the audience might perceive or assess the practitioner is of little concern to him/her. | Resolution suspended until evaluation results are known. |
| The Against the Odds Type | Conversion of stakeholders to the values and principles of community development. | Tragedy. Is characterized by a series of barriers that the practitioner hopes to overcome but is ultimately unsuccessful. | Practitioner is the central character. They are in possession of valuable knowledge. | Practice focus is process. This process is applied through relationships. The practitioner is a facilitator of change. | Characterized according to community development logic, i.e., as people to be facilitated. | Audience can see the unfolding tragedy. | The ‘invisible fate’ *i.e.*, possible intervention failure, becomes visible to the practitioner. |

* based on Gergen and Gergen 1984, 1988; Frye 1957; Ezzy 1998; Frank 2000
